# Tumor metrics imaging core labs: primer for radiologists

**DOI:** 10.1007/s00261-026-05439-8

**Published:** 2026-03-03

**Authors:** Rachana Gurudu, Dhruv Bansal, Anil Chauhan, Sree Harsha Tirumani

**Affiliations:** 1 Case Western Reserve University, Cleveland, USA; 2 Department of Medical Oncology, Endeavor Health, Chicago, USA; 3 Department of Radiology, University of Kansas Medical Center, Kansas City, USA; 4 Department of Radiology, University Hospitals Cleveland Medical Center, Case Western Reserve University, Cleveland, USA; 5 Case Western Reserve University, Cleveland, USA

**Keywords:** Tumor metrics imaging core lab, Cancer clinical trials, Imaging biomarkers

## Abstract

Imaging biomarkers have emerged as increasingly important endpoints in cancer clinical trials. Incorporating tumor metric reads as part of routine clinical reads by on-site radiologists for cancer clinical trials has several challenges which can be addressed by tumor metrics imaging core lab. Despite the operational and financial challenges inherent in establishing and maintaining tumor metrics imaging core labs, including workflow complexities, infrastructure demands, and data security considerations, these facilities confer significant advantages including accelerated trial timelines, improved regulatory compliance, and the creation of interdisciplinary research environments. Moreover, the integration of artificial intelligence within tumor metrics imaging core labs offers enhanced image analysis, predictive modeling, and improved trial efficiency. This article provides a comprehensive review of the role of tumor metrics imaging core labs in clinical trials and provides an overview of the key components involved in setting up a core lab. We will also briefly present the challenges in the successful operation of a tumor metrics imaging core lab and delve into the potential solutions, including the integration of AI tools for clinical trials.

## Introduction

The cancer clinical trials market has experienced significant growth in recent years, expected to reach a valuation of $101.86 billion dollars by 2030 [1]. To facilitate the growth and development of novel technological and pharmaceutical treatments, cancer clinical trials must involve clinicians and day-to-day practitioners in navigating stringent regulatory procedures and guidelines. Indeed, cancer clinical trials today often incorporate multiple outcomes measures to assess the safety and efficacy of an intervention or possible treatment, allowing researchers to assess the full effect of an intervention in cancer management [2]. In this regard, imaging biomarkers have emerged as increasingly important endpoints in cancer clinical trials, surpassing traditional observer-driven pattern recognition to offer greater detail about baseline disease and response to treatment over time [3]. Nearly two-thirds of the 20,000 cancer clinical trials registered in clinicaltrial.gov in the last decade use imaging-based endpoints [4]. These imaging biomarkers provide a more standardized and measurable method for assessing intervention efficacy, exploring the mechanism of action for the treatment, and ensuring a more accurate diagnosis of the enrolled cohort.

Novel cancer therapies like molecular targeted therapies and immunotherapy have unusual patterns of treatment response on imaging, necessitating the introduction of personalized treatment response criteria. The introduction of such sophisticated imaging measures and biomarkers into cancer clinical trials requires precision, accuracy, and reproducibility of response assessment, necessitating standardized protocols, expert analysis, and quality control measures across multiple research sites and countries [5]. Tumor metrics imaging core labs offer these services, assisting with clinical data intake, image analysis, data management, and quality assurance [6]. Similar imaging core lab frameworks have been applied to other specialties, including neurology, cardiology, and musculoskeletal trials using MRI and CT-based biomarkers for the analysis of lesions or ventricular function [7]. Furthermore, with the recent advent of artificial intelligence (AI) algorithms into image analysis, there is an increasing demand on tumor metrics imaging core labs to expedite cancer clinical trial reads [8, 9]. As AI algorithms demonstrate enhanced image analysis, increased operational efficiency, and improved predictive analytics, it is essential to examine the incorporation of these technological tools in tumor metrics imaging core labs, as well as in the context of oncological clinical trials [10].

Accordingly, this review paper assesses the role of tumor metrics imaging core labs in clinical trials and provides an overview of the key components involved in setting up a core lab. We will also briefly present the challenges in the successful operation of an imaging core lab and delve into the potential solutions, including the integration of AI tools into imaging core labs for clinical trials.

## Role of tumor metrics imaging core labs in clinical trials

Medical imaging plays a pivotal role in modern clinical trials, particularly in oncology, where imaging biomarkers serve as key indicators of therapeutic response and disease progression. The use of imaging biomarkers in clinical research requires consistency, accuracy, and regulatory compliance of trial data. Several meta-analyses over the years have consistently reported that local evaluation by on-site radiologists is comparable to or better than blinded independent central review (BICR) for determining progression-free survival in several randomized controlled trials [11–15]. However, there are several challenges with local evaluation by on-site radiologists for cancer clinical trials. First, incorporating tumor metric reads as part of routine clinical reads by on-site radiologists is not practical, especially given recent shortages of radiologists and the busy nature of clinical practices [16]. Second, the expertise required for performing reads in cancer clinical trials is unique and necessitates dedicated training [17]. In a study analyzing inter-reader discordances in six lung cancer trials with 1833 patients and 71 radiologists, Beaumont et al. found discrepancies between readers to be on average 32.9%, necessitating multiple reads [18]. It is to be noted that even with expert radiologists, radiological inter-reader disagreement rates of up to 20–40% are reported in several studies for various oncologic indications, and this disagreement rate has been consistent over decades [4]. Third, imaging assessment in the clinical setting by on-site radiologists can be a source of bias as the readers are unblinded to the patient’s health information. Fourth, constant monitoring of on-site radiologists for bias and errors can be impractical.

Several of these challenges can be addressed by a tumor metrics imaging core lab, which can improve the validity and consistency of imaging biomarkers in cancer clinical trials [7, 19]. Goldmacher and Raunig assessed clinical trial management and image reviews of numerous significant glioblastoma trials across each phase of drug development, involving over 4,000 patients at 900 sites [20]. They observed that collaboration between neuroradiologists and neuro-oncologists is best carried out in the setting of a tumor metrics imaging core lab and was ideal in adherence to neuro-oncology criteria [20]. A tumor metrics imaging core lab serves as a centralized hub for the standardized management and analysis of medical imaging data in clinical trials. Its primary aim is to facilitate the incorporation of imaging biomarkers, ensure interdepartmental collaboration, and maintain the generation of high-quality, reliable, quantitatively accurate, and poolable imaging datasets across multiple trial sites. These labs support the robust and regulatory-compliant evaluation of imaging-based outcomes by integrating both technological infrastructure and coordinated personnel workflows.

Imaging core labs may operate as center-based units embedded within academic hospitals or as reference-based centralized facilities managed by sponsors, CROs, or independent institutions. Separated, private facilities have developed in response to the growing demands of clinical trials and rise in medical innovation [21]. Center-based core labs primarily support local patient recruitment, acquisition harmonization, and institution-specific workflows, whereas reference-based core labs focus on centralized metric extraction, blinded independent review, and cross-site data aggregation [22]. In this review, ‘central review’ primarily refers to the latter model unless otherwise specified.

A critical feature provided by the tumor metrics imaging core labs is BICR. BICR has been shown to enhance the reliability and consistency of image results by removing bias cropping from unblinding patient information, as shown by independent image reading studies and clinical trial endpoint guidelines [22, 23]. BICR is particularly important for phase 2 and 3 single-arm or unblinded studies. Accordingly, the FDA in 2018 recommended that “clinical trial primary endpoint image readers will be blinded to a subject’s treatment assignment [22, 23].” Typical BICR involves two primary independent readers who interpret the same study subjects and an adjudicator who will reconcile any difference in opinion between the two primary readers. Furthermore, tumor metrics imaging core labs use standardized objective tumor assessment criteria such as RECIST 1.1 and RANO [24–26], which aim to reduce variability in serial assessments of lesions and are widely accepted as endpoints for the determination of treatment efficacy and patient outcome.

In addition to offering standardized, unbiased reads, tumor metrics imaging core labs enhance data oversight and regulatory compliance, and reduce the burden on medical oncologists in clinical trials (Table 1). Hersberger et al. compared the quantitative prospective response assessment by medical oncologists at a comprehensive cancer center with retrospective assessment by the newly developed tumor response assessment core (TRAC) in 49 eligible lung cancer patients across 10 cancer trials and found only moderate agreement between oncologists and TRAC. The study concluded that core labs bridge the gap in cancer clinical trials and decrease the burden on medical oncologists [27].

**Table 1:** Comparison of routine clinical reads and imaging core lab reads in cancer clinical trials

Feature	Routine clinical reads	Core lab reads
Blinding status	Unblinded, readers have access to patient health information which increases bias.	Blinded independent central review (BICR) incorporates independent readers, limiting bias from clinical information [22, 23].
Inter-reader variability	High, with some studies reporting discrepancies averaging 32.9% between readers [17].	Low, as specialized experts and adjudication of reader discordance improve reliability [22, 23].
Standardized criteria	Variability in lesion selection and measurement; often mirrors standard clinical practice.	Strict Adherence; mandated use of objective criteria such as RECIST 1.1 and RANO per regulatory guidelines [24–26].
Primary focus of workflow	Routine patient care makes clinical trial management impractical, especially with current radiologist shortages [15].	Specialized research workflows dedicated to trial timelines and regulation improve operability in core lab settings empirically [20].

This table highlights the operational and regulatory differences between local site evaluations and core lab reads in cancer clinical trials

## Key components of imaging core labs

**Fig. 1:**
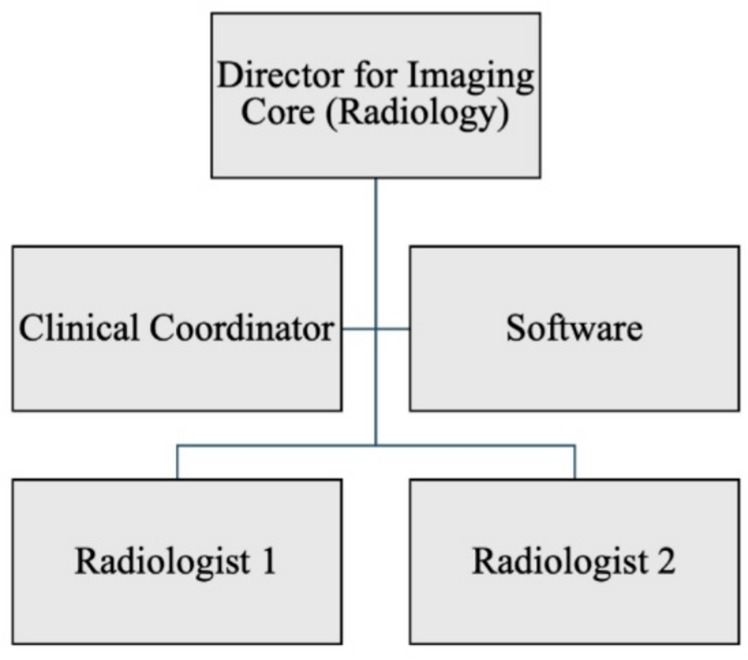
Sample imaging core lab team. Beyond the core management team and IT support depicted, essential core lab functions not visually depicted include acquisition harmonization strategies, statistical analysis planning, audit trails, and data provenance management

An ideal tumor metrics imaging core lab includes an imaging clinical trial management system that collaborates closely with the clinical trial team from the initial budgeting phase to the data transfer phase. Several commercially available clinical trial management systems are designed to manage complex workflows. Essential technological components of tumor metrics imaging core labs include cloud-based image storage systems, an image analysis platform, quality control measures and intra-institutional multi-department coordination capabilities. Successful integration of a clinical trial management system into clinical practice needs a dedicated management team, including clinical, billing, and auditing coordinators, and hospital information technology support (Fig. 1).

**Fig. 2:**
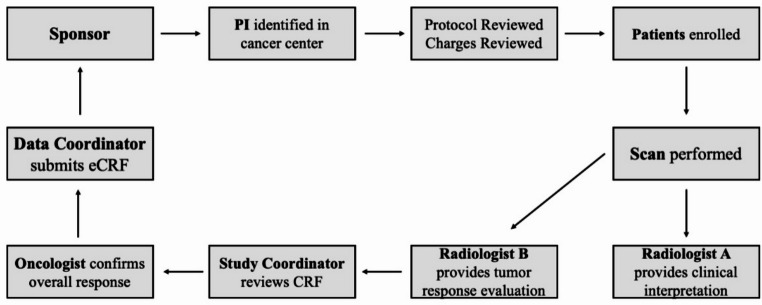
Core lab workflow

The operational workflow of an imaging core lab typically begins with the identification of a study sponsor and principal investigator, along with detailed study information on an intake form. This is followed by protocol and budgetary review, patient enrollment, lab/other tests, and treatment administration. Imaging exams are often performed using standard of care protocols unless otherwise specified by the clinical trial and transferred to the clinical trial management system with the trial identifier. Clinical trial reads are often provided independent of clinical reads, either directly by radiologists or with the help of image analysts under supervision of a radiologist. The study coordinator then reviews the corresponding electronic case report form (eCRF), after which the medical oncologist approves the overall response before submission to the sponsor (Fig. 2).

Image analysis is performed using commercially available, FDA-approved DICOM image viewers [28]. More specifically, while standard FDA-approved DICOM viewers are utilized for routine radiological reporting, dedicated image analysis platforms are required for the extraction of quantitative imaging biomarkers, radiomics, and AI-driven metrics. Data entry is conducted via electronic CRFs, which enables efficient data collection, reduces manual transcription errors, and often integrates directly with imaging software to auto-populate relevant fields. All systems must comply with U.S. Food and Drug Administration regulations, specifically 21 CFR Part 11, which governs electronic records and signatures.

Meeting the stringent requirements of 21 CFR Part 11, which apply to electronic records and signatures, tumor metrics imaging core labs must implement robust IT infrastructure beyond traditional clinical systems [23]. This necessitates the establishment of independent picture archiving and communication systems (PACS), or specialized image analysis platforms separate from hospital infrastructure to maintain data integrity [29]. Secure data pipelines are essential to support the deidentification and transfer of images while safeguarding personal health information of patients. A critical part of these pipelines are eCRFs with auto-population capabilities to reduce manual transcription errors and allow for real-time data validation. Furthermore, the adoption of common data models and controlled vocabularies is critical to ensure interoperability across institutions with heterogeneous ontologies and imaging descriptors [30] (Table 2).

**Table 2:** Tumor metrics imaging core lab IT infrastructure and regulatory checklist for radiologists

Category	Requirement
System isolation	Independent research PACS or analysis platform for image retrieval and storage.
Data security	Secure and encrypted data pipelines for image transfer and communication.
De-identification	Automated de-identification tools for images to protect personal health information.
Electronic records	21 CFR Part 11 compliant electronic signatures.
Workflow integration	Integrated eCRF with auto-population capabilities to reduce manual errors and ensure validation with trial database.

Beyond data integrity, trials should have robust corrective and preventive action (CAPA) plans to maintain quality assurance. These plans, triggered by the identification of non-compliant errors, aim to prevent loss of trial integrity. Corrective action, for technological issues or protocol deviations, may involve flagging scans and notifying the principal investigator, identifying the error through validation in the eCRF, mandatory adjudication by another reader, or repeat scans in compliance with IRB requirements [31]. Preventative action involves changing standard operating protocols, enhancing eCRF auto-population features to eliminate errors, or implementing re-training based on audit trends.

To ensure consistency and reproducibility, tumor metrics imaging core labs develop and maintain SOPs that govern imaging acquisition and analysis protocols across all trials. The following template outlines the essential domains these protocols must cover to meet the demands of oncology clinical trials.

### Core lab SOP template

- Imaging Acquisition & Analysis.
  - Mandate the use of standardized objective tumor assessment criteria (e.g. RECIST 1.1, RANO) to reduce variability in serial assessments.
  - Define protocols for image interpretation, specifying readers.
- Image Tracking & Data Transfer.
  - Establish procedures for integrating trial numbers and de-identification prior to exporting or transferring data.
  - Standardize the identification of study information on intake forms to streamline workflows.
- Data Validation & Quality Assurance.
  - Implement annual audits for each active study, including the development and annual review of a quality policy manual [31].
  - Review corrective plans for any identified deficiencies in weekly core lab meetings, guided by input from lab managers and directors.
- CRF Completion & Data Management.
  - Use eCRFs to reduce manual transcription errors and integrate with imaging software for auto-population.
  - Ensure adherence with 21 CFR Part 11 guidelines and trial regulation.
- Long-Term Archiving.
  - Utilize robust and secure IT infrastructure for image storage and retrieval.
  - Maintain secure data pipelines to support archiving across multiple trial sites for larger oncology trials.

## Challenges

While tumor metrics imaging core labs play a crucial role in clinical trials, the successful integration of a core lab into clinical practice presents several challenges that can impact their performance, efficiency, and sustainability.

### Costs

The upfront cost of setting up and maintaining tumor metrics imaging core labs is a significant barrier to their establishment. The diverse funding sources for radiological and oncological studies often limit the size and operational capacity of these labs, reducing their ability to provide efficient, real-time reads independent of the clinical trial team. Hersberger et al. noted, for example, a lack of uniformity in workflows at different institutions because of budgetary constraints due to variable grant restrictions [27].

Revenue models for core laboratories vary across institutions and depend on whether the trial is investigator-initiated or industry-sponsored. Some institutions may restrict core laboratories from operating as profit-generating entities, aligning billing rates with National Institutes of Health (NIH) guidelines or industry standards. With broader financial constraints already impacting clinical trials and drug development, the need for separate and stable funding for tumor metrics imaging core labs remains a critical consideration, necessitating self-sustaining revenue models [32].

### Introduction of complex workflows

Cancer clinical trial workflows often require intricate coordination across multiple stakeholders. Many academic institutions in the United States provide cancer clinical trial reads separate from standard-of-care clinical reads. This requires establishing an independent workflow. For instance, ordering research scans typically involves integrating trial numbers and patient identifiers while ensuring that appropriate de-identification is performed prior to data export. This necessitates a thorough understanding of the trial design, patient recruitment procedures, and data handling requirements. Missteps in these workflows can result in delays or the introduction of bias and compromise data integrity [33].

Coordination between radiologists, trainees, image analysts, managers, and research staff requires consistent communication and familiarity with research-specific protocols [34]. Many involved personnel may have varying levels of clinical and trial-specific experience, making standardization challenging. Radiologists recognize the need for specialized clinical trial experience and training, which differs from traditional clinical practice, as it can be time-intensive and challenging to implement frequently in a core lab setting [16].

### Data management and security

The handling of imaging data introduces a separate set of challenges, particularly with regards to standardization and security. Errors in the de-identification process for DICOM images can pose a significant threat to patient confidentiality and introduce bias into trial outcomes, necessitating strict adherence to best practices [34]. Furthermore, integrating imaging systems with clinical trial databases requires a robust IT infrastructure and secure data pipelines to support image transfer, storage, and retrieval across multiple sites. Many institutions have recently established dedicated cybersecurity teams to enhance these processes, ensuring compliance with regulatory standards and safeguarding sensitive data [35].

### Data quality

Despite standardized response criteria for imaging endpoints, variability in image interpretation among readers remains a significant issue. Differences in how lesions are measured, which lesions are selected, or whether subtle changes are deemed significant may lead to discrepancies that necessitate adjudication processes, delaying study timelines. In a study involving 1,053 patients, Margolis and Ruchalski reported that, overall, imaging core labs yield more accurate interpretations and demonstrate less overestimation compared with routine clinical assessments [36].

Moreover, variation in imaging equipment and software — including vendor-specific differences in camera systems, reconstruction algorithms, and pricing models — can affect image quality and consistency across sites. Imaging core labs with expertise in advanced methods, such as machine learning or 3D tumor mapping, may also require specialized software that diverges from institutional norms, further complicating quality assurance efforts [35, 37]. Data quality technical quality control procedures typically include verification of image series completeness, adherence to acquisition parameters (e.g. slice thickness, contrast timing, reconstruction kernels), artifact detection, and validation of DICOM metadata consistency prior to analysis. These methods can improve reproducibility in image analysis, which is critical in clinical trial reads and advanced radiomics analysis [38, 39].

## Cost-benefit analysis of tumor metrics imaging core labs

Implementing a tumor metrics imaging core lab involves significant initial investments, encompassing infrastructure, personnel, and technology. Revenue models for imaging core lab services vary and can include:

- Annual Subscription Fees: Institutions may offer bulk usage plans with annual fees, providing access to equipment and services over a set period. For example, some imaging core labs provide annual subscription models that grant unlimited or tiered access to imaging services.
- Per-Timepoint Charges: Fees are assessed based on specific timepoints in a study, such as baseline, mid-study, and end-of-study imaging sessions. This model enables cost allocation that is aligned with study milestones.
- Per-User or Per-Department Rates: Charges are determined by the number of users accessing the imaging services, which can be beneficial for studies with varying team sizes. Departments can also fund specific FTEs of roles required for the successful operation of an imaging core lab:
  i. Lead Image Analyst: Responsible for the primary execution of tumor metrics and data quality under radiologists.
  ii. Research/Billing Coordinator: Manages financial workflows, including trial billing and lab budgeting.
  iii. IT Support: Provides maintenance for trial databases, PACS, eCRFs, and other electronic systems in compliance with 21 CFR Part 11.
  iv. Core Lab Manager or Director: Provides high-level oversight of operating procedures, quality manuals, and meetings with contracted organizations or institutions.

These revenue models can pose financial challenges, particularly for smaller institutions or investigator-initiated trials with limited budgets. It may take 1–3 years for the system to become self-sustaining, with initial system support being essential during this period. These initial years, often described as a financial “valley of death,” require high upfront costs for infrastructure and specialized personnel before steady revenue from active clinical trials comes in [40]. To alleviate this burden, tumor metrics imaging core labs must secure initial system support or institutional bridge funding. Presenting detailed financial analyses to demonstrate how the lab will eventually become self-sustaining through the revenue models above can increase the likelihood of senior leadership support [41]. Securing bridge funding requires demonstrating the lab’s ability to attract high impact, multi-site trials that increase the institution’s research profile. Additionally, implementing a subsidized model for investigator-initiated trials can secure long-term funding, while aligning with NIH billing or industry standard guidelines [42].

Despite these challenges, the formation of tumor metrics imaging core labs presents valuable opportunities for institutions that house cancer clinical trials. As outlined below, these labs can enhance data consistency, improve operational efficiency, and foster collaborative research efforts:

1. Accelerated Trial Execution: Through a development of SOPs that emphasize quality assurance and blinded, independent reads, tumor metrics imaging core labs offer improved clinical trial workflow when compared to local site radiological reads, producing more cost-effective and timely response assessments [37]. By standardizing imaging protocols and centralizing data management, tumor metrics imaging core labs streamline workflows, reducing delays associated with image acquisition and analysis across multiple sites. This standardization ensures uniformity in imaging results, which is crucial for multi-site trials.
2. Enhanced Data Analysis with AI: Tumor metrics imaging core labs employ advanced technologies, including artificial intelligence (AI) and machine learning algorithms, to improve image interpretation accuracy and consistency. AI-driven tools can detect patterns and anomalies with high precision, aiding in the assessment of tumor growth kinetics and other critical biomarkers [43, 44]. Through ongoing data collection and analysis, tumor metrics imaging core labs have the potential to create data repositories that can improve research output and be used to develop predictive models for key conditions. Over time, imaging data collected from trials can be used to predict tumor growth kinetics and model the effect of radiation pre-trial [45].
3. Expert Reads and Interdisciplinary Collaboration: Access to a centralized pool of specialized radiologists ensures consistent and high-quality image assessments, minimizing variability and potential biases in interpretation [46]. In addition, by incorporating traditional trial leadership and medical oncologists with radiologists and core lab faculty, tumor metrics imaging core labs promote interdisciplinary collaboration and innovation in the field of cancer imaging between physicians, scientists, and industry. Indeed, interdisciplinary perspectives and members in clinical trial management ensure effective conflict resolution, adequate time for collaboration, and the proper use of technology, among other benefits [47]. Additionally, implementing a subsidized model for investigator-initiated trials can further enhance collaboration by alleviating financial constraints, thereby fostering a more inclusive and cooperative research environment.
4. Regulatory Compliance and Multi-Trial Management: Tumor metrics imaging core labs adhere to stringent regulatory standards, such as ICH-GCP and HIPAA, ensuring that imaging data management meets necessary legal and ethical requirements. Their processes involve standardized imaging acquisition protocols, regular quality control checks, secure data storage solutions, and comprehensive audit trails for documentation [48]. In addition, tumor metrics imaging core labs provide a self-sustaining model that can be used in numerous clinical trials to come, allowing for ongoing trial management and efficiency in managing multiple trials at an institution at once. In a study of imaging core labs in 5 NCTN trials, Binzel et al. found that real-time quality assurance reports and timely feedback from core labs bring critical awareness to non-compliant errors, increasing consistency over the course of multiple trials of imaging analysis [49].

## AI in clinical trial management

While radiomics can be derived using handcrafted feature extraction independent of artificial intelligence, AI-based methods increasingly automate segmentation, feature selection, and predictive modeling within imaging core labs. Thus, the incorporation of artificial intelligence (AI) into tumor metrics imaging core labs presents a transformative opportunity to enhance cancer clinical trial workflows. AI algorithms can automate steps such as scan quality control and image de-identification, improving workflow efficiency and compliance with regulatory standards for centralized imaging [50]. A primary practical benefit is automated metadata capture, allowing for the extraction and validation of image header information such as scanning parameters and contrast timing [51]. In turn, AI models are able to mask identifiers to comply with privacy regulation, consistently export data, and maintain data security through file protection [52]. By replacing manual data entry, tumor metrics imaging core labs can identify protocol deviations in real-time and ensure conformity to imaging protocols across sites, accelerating trial setup and execution timelines [53].

AI-assisted cancer clinical trial reads offer significant advantages in the image interpretation phase of clinical trials, particularly in the oncologic imaging. Deep learning algorithms can support lesion detection, tissue segmentation, and semi-automated measurement of tumor burden, substantially reducing the manual annotation time required by radiologists [54]. In an analysis of a deep learning model on 6,716 lung cancer cases, Ardila et al. found that the algorithm outperformed radiologists, reducing false positives and negatives, thereby optimizing lung cancer screening [54]. These tools help mitigate reader fatigue and reduce inter-reader variability, two known sources of error in multicenter imaging trials [55]. By incorporating AI into independent central reads, tumor metrics imaging core labs can improve efficiency while maintaining accuracy and blinding integrity.

AI algorithms support improved protocol design through the integration of quantitative image metrics that extend beyond traditional size-based endpoints. Radiomics and quantitative textural analysis techniques extract high-dimensional features from imaging datasets to characterize tumor heterogeneity, vascularity, and treatment response [56, 57]. Radiomics incorporates volume of interest identification, where specific anatomical regions are targeted for study, segmentation, or the creation of boundaries with deep learning algorithms, and feature extraction and qualification, which harvests quantitative data regarding tissue shape or texture, enabling multilevel analysis and diagnosis [51]. In a radiomic analysis of 1,019 patients, analyzing 440 features, radiomics was found to have prognostic power in patients with lung and head and neck cancers, improving clinical outcomes [58]. These features can be integrated into trial endpoints to enhance stratification or facilitate early response evaluation, providing more sensitive markers of therapeutic efficacy. Deep learning methods also model tumor growth kinetics and simulate treatment response trajectories, aiding in adaptive trial designs or dose optimization [58]. As tumor metrics imaging core labs have access to structured imaging data, the opportunity to incorporate predictive modeling into protocol development increases.

In the data analysis phase, AI enables enhanced data integration and pattern recognition, contributing to more robust trial analytics. Commercially available platforms allow for structured data capture during image interpretation, enabling automated audit trails and integration with clinical trial data management systems [53]. Machine learning models trained on large-scale, annotated imaging data may help predict clinical outcomes, informing future trial designs and stratification strategies [59]. Centralized data capture systems and harmonized reporting tools also improve reproducibility and regulatory compliance, especially in multi-trial or multi-site settings. As tumor metrics imaging core labs develop internal imaging repositories, their contributions to innovation, monitoring, and multi-trial analysis are likely to grow as well.

Beyond clinical and analytical applications, AI enhances administrative operations within tumor metrics imaging core labs. AI-driven electronic case report forms (eCRFs) automate data entry, reducing transcription errors and ensuring consistency across trial sites. Real-time validation features in eCRFs can instantly flag discrepancies, such as patient data inconsistencies, streamlining data management processes.

In summary, integrating AI into tumor metrics imaging core labs not only advances clinical and analytical capabilities but also streamlines administrative processes, enhances communication, and supports oncologists in patient interactions, thereby contributing to more efficient and effective cancer clinical trials.

## Conclusion

Tumor metrics imaging core labs play a critical and increasingly indispensable role in the success and integrity of modern clinical trials, particularly in oncology. As trials become more complex and multi-institutional, tumor metrics imaging core labs offer the standardization, quality assurance, and expert oversight necessary to ensure accurate, reproducible, and unbiased imaging data. Through blinded independent reads, adherence to standardized criteria such as RECIST and RANO, and reduction of inter-reader variability, imaging core labs significantly enhance the reliability of trial outcomes while alleviating burdens on site-based radiologists and oncologists.

Despite the operational and financial challenges inherent in establishing and maintaining tumor metrics imaging core labs, including workflow complexities, infrastructure demands, and data security considerations—these facilities confer significant advantages. These include accelerated trial timelines, improved regulatory compliance, and the creation of interdisciplinary research environments. Moreover, the integration of artificial intelligence within tumor metrics imaging core labs offers enhanced image analysis, predictive modeling, and improved trial efficiency.

## Data Availability

No datasets were generated or analysed during the current study.
